# Nanosilver-Functionalized Hybrid Hydrogels of Carboxymethyl Cellulose/Poly(Vinyl Alcohol) with Antibacterial Activity for Prevention and Therapy of Infections of Diabetic Chronic Wounds

**DOI:** 10.3390/polym15234542

**Published:** 2023-11-27

**Authors:** Nádia S. V. Capanema, Alexandra A. P. Mansur, Sandhra M. Carvalho, Talita Martins, Maysa S. Gonçalves, Rafaella S. Andrade, Elaine M. S. Dorneles, Letícia C. D. Lima, Érika L. F. C. de Alvarenga, Emanuel V. B. da Fonseca, Marcos Augusto de Sá, Andrey P. Lage, Zelia I. P. Lobato, Herman S. Mansur

**Affiliations:** 1 Center of Nanoscience, Nanotechnology, and Innovation—CeNano^2^I, Department of Metallurgical and Materials Engineering, Federal University of Minas Gerais, UFMG, Belo Horizonte 31270-901, Brazil; nsvnadia@gmail.com (N.S.V.C.); alexandramansur.ufmg@gmail.com (A.A.P.M.); sandhra.carvalho@gmail.com (S.M.C.); martins.talita2009@gmail.com (T.M.); 2 Departamento de Medicina Veterinária, Universidade Federal de Lavras, UFLA, Lavras 37200-000, Brazil; maysaserpa@gmail.com (M.S.G.); rafaellabambuii@gmail.com (R.S.A.); elaine.dorneles@ufla.br (E.M.S.D.); 3 Department of Morphology, Institute of Biological Sciences, Federal University of Minas Gerais, UFMG, Belo Horizonte 31270-901, Brazil; leticia.cristina93@gmail.com (L.C.D.L.); samarcos2005@yahoo.com.br (M.A.d.S.); 4 Department of Natural Sciences, Universidade Federal de São João Del-Rei, UFSJ, São João Del-Rei 36301-160, Brazil; erika.fisio@ufsj.edu.br (É.L.F.C.d.A.); vitorfonseak@gmail.com (E.V.B.d.F.); 5 Departamento de Medicina Veterinária Preventiva, Federal University of Minas Gerais, UFMG, Belo Horizonte 31270-901, Brazil; aplage11@outlook.com (A.P.L.); ziplobato@gmail.com (Z.I.P.L.)

**Keywords:** nanosilver-functionalized hydrogels, antibacterial wound dressings, carboxymethyl cellulose-poly(vinyl alcohol)-based hydrogels, polymer-silver nanoparticles hybrid nanocomposites, polysaccharide-based diabetic chronic wound dressing

## Abstract

Diabetic foot ulcers (DFUs) are considered one of the most severe chronic complications of diabetes and can lead to amputation in severe cases. In addition, bacterial infections in diabetic chronic wounds aggravate this scenario by threatening human health. Wound dressings made of polymer matrices with embedded metal nanoparticles can inhibit microorganism growth and promote wound healing, although the current clinical treatments for diabetic chronic wounds remain unsatisfactory. In this view, this research reports the synthesis and characterization of innovative hybrid hydrogels made of carboxymethyl cellulose (CMC) and poly(vinyl alcohol) (PVA) chemically crosslinked by citric acid (CA) functionalized with silver nanoparticles (AgNPs) generated in situ using an eco-friendly aqueous process. The results assessed through comprehensive in vitro and in vivo assays demonstrated that these hybrid polymer hydrogels functionalized with AgNPs possess physicochemical properties, cytocompatibility, hemocompatibility, bioadhesion, antibacterial activity, and biocompatibility suitable for wound dressings to support chronic wound healing process as well as preventing and treating bacterial infections. Hence, it can be envisioned that, with further research and development, these polymer-based hybrid nanoplatforms hold great potential as an important tool for creating a new generation of smart dressings for treating chronic diabetic wounds and opportunistic bacterial infections.

## 1. Introduction

Diabetes mellitus (DM) is recognized as a severe metabolic disorder mainly characterized by permanent uncontrolled high levels of blood glucose, i.e., hyperglycemia, that may be from various origins [1,2]. Although transient hyperglycemic conditions can occur, such as in gestational diabetes, type 1 and type 2 diabetes are long-term chronic conditions that have emerged as one of the most serious and prevalent diseases of our time [1]. Regrettably, the global incidence of DM is rising, and almost 50% of adults are unaware of their diabetes conditions. There is an estimated 10.5% prevalence in 20–79 year olds in 2021 (~500 million), increasing to 12.2% (~800 million) in 2045 [3]. Complications in diabetic patients often include cardiac and renal disorders and ocular, neurological, and vascular issues, resulting in a significant global health burden [1,2,3,4,5,6]. Neuropathy and vasculopathy are particularly linked to developing severe diabetic chronic wounds, most notably foot ulceration, termed diabetic foot ulcers (DFUs). Unfortunately, DFUs usually progress very rapidly, requiring extensive treatment and becoming a long-term complication. DFUs present a significant risk of amputation, morbidity, and even death due to the lack of efficient therapies, posing an important challenge to the patient and the healthcare system worldwide [1,2,3,4,5,6,7,8,9].

Because of the numerous specificities of the diabetic wound environment, which include hyperglycemia, oxidative stress, decreased angiogenesis, deficiency of growth factors, and other aspects, conventional treatments are often unsuccessful. Moreover, diabetic wounds are associated with a significant increase in microbial infection risk that can lead to chronic inflammatory conditions [1,3,4,7,8,9]. In this view, innovative topical therapy strategies are highly needed to build an appropriate microenvironment for promoting diabetic chronic wound (DCW) healing. Thus, diabetic wound therapeutic approaches such as gazes, foams, films, hydrocolloids, hydrogels, scaffolds, membranes, injectable systems, and others have been investigated. Still, no ideal dressing exists for all kinds of wounds [1,2,3,4,5,6,7,8,9].

Nonetheless, some dressing characteristics should be pursued for chronic wounds, such as exudate excess removal, wound debridement, balanced moisture maintenance, gas exchange and permeability, infection prevention, mechanical protection, adherence, and a low frequency of dressing change. Hence, an effective wound dressing should be responsive to changes in these dynamic wound microenvironment conditions [1,2,3,4,5,6,7,10,11].

In this sense, a new generation of promising multifunctional biomaterials relying on polymer hydrogels has emerged. They frequently amalgamate various components into “smart” hybrid matrices focusing on novel therapeutic strategies and with superior properties aimed at fulfilling most of the crucial requirements for diabetic wound treatment [9,10,11,12,13,14,15,16,17].

As a background knowledge for this multidisciplinary area of research, hydrogels can be defined as crosslinked hydrophilic polymer macromolecular chains from natural or synthetic sources, forming chemically stable 3D networks that can absorb a large quantity of water and hold in their interstitial structures, leading to a water-swollen state [18,19,20,21,22,23]. Crosslinked polymer networks may be established via covalent bonding, electrostatic, and physical interactions involving chemical groups attached to the hydrophilic polymer chains. The types of polymers and crosslinking combinations give rise to virtually limitless possibilities for producing hydrogels with a broad range of properties designed for numerous applications. In particular, considering skin tissue engineering tailored to wound healing applications, in addition to the required biocompatibility, hydrogels can readily adapt to irregularly shaped wounds combined with the high ability to absorb exudates, promote moisture balance at the wound site, and aid in autolytic debridement [9,10,11,12,13,14,15,16,17,24,25].

Moreover, hydrogels can be tailored to provide superior physicochemical, mechanical, and biochemical properties and features compared to other wound dressing alternatives. They can improve adhesiveness, injectability, self-healing capability, biocompatibility, drug release property, cell-delivery capacity, antibacterial activity, antioxidant capacity, and cell hosting capacity, which are extremely important for diabetic wound healing [7,8,9,10,11,12,13,14,15,16,17]. Thus, these characteristics make hydrogels the most attractive strategy for developing multifunctional “smart” dressings for treating chronic diabetic wounds [13,14,15,16,17]. These “smart hydrogels” can be produced by amalgamating several components, usually natural polymers (e.g., polysaccharides and polypeptides), synthetic polymers, and chemically modified blends, often called hybrid hydrogels [10,11,18,19,20,21].

Generally, naturally-derived polymers (or biopolymers) are more prone to mimic the biophysical and biochemical complexity of skin tissues than synthetic artificial polymers [26,27]. Among naturally sourced polymers, a semi-processed polysaccharide, carboxymethyl cellulose (CMC), has been selected for producing hydrogels as skin substitutes and wound dressings relying on its intrinsic biocompatibility, biodegradability, and more effective adherence to wound sites while exhibiting high exudate absorbency [23,25,26,27]. CMC has been shown to enhance wound healing through in vivo models. Moreover, CMC significantly promotes the regulation of transdermal water loss and exhibits a prominent water-absorbing ability, which minimizes moisture loss in the wound microenvironment. CMC and [23,26,27,28]. Although CMC hydrogels can knowingly contribute to enhancing the compatibility with skin, favoring the chronic wound healing process (e.g., hemostasis, blood clotting, inflammation, cell adhesion, and cellular migration), they do not fulfill all requirements for mimicking skin tissue. For instance, CMC hydrogels are inappropriate for tunable mechanical properties (e.g., stiffness, flexibility, and elasticity), considering the 4 stages of the healing progression cascade [23,28,29]. Thus, using polysaccharide-based hydrogels (e.g., CMC) requires their properties to be improved to assign new functions and features important to assist in wound healing, which can be achieved by combining with other biopolymer or synthetic polymers and applying chemical functionalization. Unfortunately, such a wound dressing platform is not available yet, requiring further developments in the field [23,28,29].

In this view, hydrogels and combined composite materials of CMC with poly(vinyl alcohol) (PVA) have increased interest over recent years predominantly because of their relative facile synthesis and chemical modifications amalgamating various properties with morphological structural features. As a result, notable improvements have been verified in the mechanical strength, thermochemical stability, and flexibility of the biopolymeric network of CMC when it is blended and chemically crosslinked with PVA polymer [30,31]. Therefore, PVA, a broadly available hydrophilic synthetic polymer, has been one of the most frequent choices associated with CMC to produce hydrogel hybrids for biomedical applications. CMC and PVA are miscible and compatible hydrophilic polymers mostly driven by hydrogen bonding formation [30]. Moreover, they are nontoxic and environmentally friendly polymers suitable for many advanced biomedical engineering applications. Consequently, the biopolymer-based blends, (nano)composites, and hybrids have been subjected to intensive research because they offer an attractive integrated set of multiple functionalities and properties to mimic the skin tissue characteristics, facilitating the wound healing cascade process and tissue repair [30,31,32].

Nonetheless, despite the undisputable advantages of polymer-based hydrogels for wound dressings, as highlighted above, they often lack sufficient efficiency to avoid antimicrobial infections in DFUs. Unfortunately, because of the frequent hyperglycemic conditions of the wound site, most diabetic wounds are prone to bacterial infections, as they provide a favorable environment for the growth of microorganisms. Hence, infected chronic wounds represent an even more complex and non-trivial problem that seriously endangers the health and life of diabetic patients [33,34,35]. Additionally, the common much longer healing period of infected DFUs leads to the continuing use of antibiotics, which may increase drug resistance possibility and restrict therapeutic drug alternatives. Ultimately, serious systemic health problems may arise from infected wounds, especially if inaccurately treated, posing the greatest risk of morbidity with lower member amputation and the main cause of death of diabetic patients [34,35,36].

Besides using antibacterial biomolecules and polymers (e.g., antibiotics, chitosan, and polylysine) to face this challenge, hydrogels and hybrids can be functionalized with inorganic nanoparticles (nano-functionalized), such as nanometals, incorporated into the polymer-based network to augment the antibacterial activity for improving chronic wound healing [33,35,36,37]. Silver nanoparticles (AgNPs or “nanosilver”) have been the preferred choice among inorganic nanomaterials. They attract great interest in soft tissue engineering applications, relying on their antibacterial, antifungal, and antiviral properties associated with their unique physicochemical and biological characteristics. Therefore, AgNPs have been incorporated to render antibacterial properties to hydrogels focused on chronic diabetic wound applications and wound management products. Moreover, AgNPs have been demonstrated to promote antibacterial response against a broad spectrum of microorganisms, effective against Gram-negative and Gram-positive bacteria, confirmed against ca. 650 bacterial strains, superior to conventional antibacterial drugs.

Although not fully elucidated, the most widely accepted mechanism of AgNP as an antibacterial agent is assigned to generate free radical forms of oxygen, damaging the cell walls of bacteria or their subcellular structures [33,34,35,36,37].

Introducing uniformly distributed AgNPs into the hydrogel matrices can be difficult due to the subtle chemical stability of the metallic nanocolloids in aqueous media, which tends to agglomerate, forming clusters. Consequently, it can lead to the undesirable reduction of the antibacterial activity and a harmful increase in cytotoxicity with collateral side effects in patients. To avoid this drawback, the design and development of hybrid biopolymer-based hydrogels with embedded AgNPs eliciting uniformity and chemical and structural stabilities are highly demanded to be applied for chronic wound dressings [33,34,35,36]. Albeit advancing at an increasingly fast pace, with several reports investigating the potential use of polymer-based hydrogels with AgNPs embedded for antibacterial activity, there is still a lack of reliable, effective, and affordable treatment options for DFUs. In addition, wound infections very often pose a serious challenge to diabetic wound healing, especially drug-resistant bacteria (e.g., antibiotics) and multiple microorganism infections.

Hence, in this research, innovative hybrid hydrogels were designed and developed with tailored physicochemical properties achieved by combining carboxymethyl cellulose biopolymer and poly(vinyl alcohol) chemically crosslinked with citric acid (CA) using a green aqueous chemical process (i.e., no toxic crosslinker such as glutaraldehyde and epichlorohydrin, and mild temperatures). Moreover, they were functionalized with silver nanoparticles through in situ reduction by the polymer functional groups, without an external toxic reducing agent (e.g., NaBH_4_, etc.), based on an entirely sustainable and biocompatible chemical route. These hybrid polymer-based nanocomposites were extensively characterized through several morphological and spectroscopic techniques associated with in vitro and in vivo biological analyses. These hybrid hydrogels demonstrated physicochemical properties, cytocompatibility, and antibacterial activity in vitro combined with biocompatibility using mouse in vivo models well suited for potential applications in favoring chronic diabetic wound healing.

## 2. Materials and Methods

### 2.1. Materials

Sodium carboxymethyl cellulose salt (CMC, degree of substitution 0.81 ± 0.05, and molar mass, Mw, 700 kDa), poly(vinyl alcohol) (PVA, Mw = 85–124 kDa and degree of hydrolysis 99.3–100%), and citric acid (CA, ≥99.5%) were purchased from Sigma-Aldrich (St. Louis, MO, USA). Silver nitrate (≥99.9%) was supplied by Synth (São Paulo, SP, Brazil). All materials were used as received without further purification. All solutions, unless specified otherwise, were prepared with deionized water (DI water, resistivity of 18 MΩ·cm, Simplicity^®^ Water Purification System, Merck Millipore, MA, USA) at room temperature (RT, 23 ± 2 °C).

### 2.2. Synthesis of Hybrid Polymer Hydrogels CMC/PVA/CA

In this study, the synthesis of hybrid hydrogels was based on a recent report published by our research group [10]. Thus, only the most important procedures and protocols were summarized and presented here to avoid redundancy. In brief, hybrid hydrogels using carboxymethylcellulose (CMC solubilized as a clear solution, 80% m/m of hydrogel) and poly(vinyl alcohol) (PVA solubilized as a clear solution, 20% m/m of hydrogel) were synthesized using citric acid (CA, CA/[CMC + PVA] = 25%, m/m) as a nontoxic and eco-friendly chemical crosslinking agent. The process was performed at mild temperatures (drying at 40 °C/24 h and crosslinking reactions at 80 °C/24 h). After cooling, the hydrogel membranes were immersed in DI water for 24 h (RT) to remove unbound chains and the unreacted crosslinking agent, then dried at 40 ± 2 °C/24 h. These samples were referred to as CMC/PVA/CA or CA25. As a reference, samples without citric acid (CA) were also prepared and identified as CMC/PVA or CA0. It should be noted that the full solubilization of precursor polymer aqueous solutions of PVA and CMC depends on several aspects, including the degree of hydrolysis (PVA) and the degree of substitution (CMC), molecular mass, and pH, among other parameters.

### 2.3. Synthesis of Nanosilver-Functionalized Hybrid Polymer Hydrogels

In this study, AgNPs were prepared using water as an environmentally benign solvent and the same mixture of CMC/PVA polymer solutions (mass ratio of 80:20 CMC:PVA), which was the base for obtaining the pristine crosslinked hydrogels that were presented in the previous Section 2.2. To achieve hydrogels with different silver nanoparticle concentrations incorporated, silver nitrate solutions (10 mL) were added dropwise to CMC-PVA solution under magnetic stirring at Ag concentrations (Ag (m/m %) = [Ag]/[CMC + PVA]) of 0.15% (CMC/PVA_Ag015), 0.30% (CMC/PVA_Ag030), and 0.60% (CMC/PVA_Ag060) of Ag. These systems were heated to boiling (95 ± 5 °C) to produce in situ silver nanoparticles (i.e., external reducing agent), mostly promoted by the chemical reduction of Ag^+^ by polymer hydroxyl groups [37].

After boiling, the reaction mixture was maintained under continuous moderate magnetic stirring for 15 min. After that, the suspensions of silver nanoparticles (CMC/PVA_AgNPs) were let to cool down at RT and stored at 6 ± 2 °C for further use.

The nanosilver-functionalized hybrid hydrogels were obtained upon the addition of citric acid (CA/CMC + PVA = 25%) to the suspensions of AgNPs and the same thermal treatment of the previous hydrogels (40 °C/24 h + 80 °C/24 h) after casting. Table 1 summarizes sample identification and composition.

### 2.4. Characterization of Colloidal AgNPs and Hydrogels

Ultraviolet–visible (UV–vis) spectroscopic analyses of colloidal Ag nanoparticles and nanosilver-modified hydrogels were performed using Lambda EZ-210 (Perkin-Elmer, Waltham, MA, USA) in transmission mode in a wavelength range between 700 and 370 nm.

Nanostructural characterization of the AgNPs was based on the images obtained using a Tecnai G2–20-FEI (FEI Company, Hillsboro, OR, USA) transmission electron microscope (TEM) at an accelerating voltage of 200 kV. The samples were prepared for TEM analyses by lacing a drop of dilute CMC/PVA_AgNPs colloidal dispersion onto holey carbon-coated copper grids (Electron Microscopy Sciences) and allowing them to dry overnight at RT. The dimensions of silver nanometals were assessed based on the images captured via TEM by measuring at least 25 randomly selected sites, where their average size and size-distribution data were obtained using an image processing program (ImageJ, version 1.80, public domain, National Institutes of Health).

Fourier transform infrared spectroscopy (FTIR) analysis was recorded with a Nicolet 6700 (Thermo Fisher Scientific Inc., Waltham, MA, USA) spectrometer with background subtraction. The FTIR spectra of the hybrid hydrogel membranes were obtained using attenuated total reflectance (ATR, 4000–750 cm^−1^, 32 scans, and 4 cm^−1^ resolution).

Thermal analyses were performed using thermogravimetric (TGA) and differential scanning calorimetry (DSC) techniques (SDT Q-600 instrument, TA Instruments Co., Milford, MA, USA). Hydrogel films of 1.5 ± 0.3 mg were tested at a 10 °C/min heating rate from RT to 500 °C.

The hydrogel samples were placed into a top-open platinum pan using an empty cup as the reference. The TGA/DSC analyses were performed under a continuous flow of nitrogen gas (dry, at 50 mL/min).

X-ray photoelectron spectroscopy (XPS) analysis of CMC-PVA_AgNPs was performed using Mg-Ka as the excitation source (Amicus spectrometer, Kratos). XPS analyses were performed in hydrogels. The positions of peaks were corrected based on C 1 s binding energy (285.0 eV).

For swelling degree (SD) measurement and gel fraction (GF) assessments, the membranes were cut into round samples with a diameter of 5 cm, dried at 40 ± 2 °C for mass stabilization, and weighted (W_0_, initial mass). Then, the hydrogels (replicates, n ≥ 3) were placed in 10.0 mL DI water at RT. After 24 h, the hydrogel was removed from the solution, gently wiped to remove excess liquid on the sample surface, and weighed (W_s_, swollen mass). In the sequence, samples were dried at 40 ± 2 °C until reaching mass stabilization, and the final weight (W_f_, final mass) was recorded.

Based on the literature reports [10,11,23,38,39,40,41], weight measurements obtained for each stage of the process were used to calculate the swelling degree and gel fraction of hydrogels using Equations (1) and (2), respectively. Each measurement was performed in triplicate for both the swelling degree and solubility tests. The results of SD and GF were presented as the average ± standard deviation (n ≥ 3).

SD (%) = ((W_s_ − W_0_)/W0) × 100% (1)

GF (%) = ((W_0_ − W_f_)/W0) × 100% (2)

### 2.5. In Vitro Characterization of CMC–PVA_AgNP Hybrid Membranes

#### 2.5.1. Cytotoxicity

Human embryonic kidney (American Type Culture Collection (ATCC^®^) CRL-1573™, HEK 293T) cells were provided by the Federal University of Minas Gerais (UFMG). Human malignant melanoma (A375, ATCC^®^ CRL-1619™) was purchased from the Brazilian Cell Repository (Banco de Células do Rio de Janeiro: BCRJ, Brazil). Fibroblasts isolated from the lung tissue (MRC5) were provided by the Fundação Ezequiel Dias (FUNED, Profa. Luciana M. Silva). The initial cytocompatibility bioassays of the hydrogels were conducted according to the most widely accepted international standard (ISO 10993-5:2009 [42]), applied for the preliminary biological evaluation of medical devices. All cell manipulations were performed with sterile items and media and were conducted aseptically in a laminar flow hood. Before the experiments, the samples were sterilized by a UV radiation source for 30 min on each side. The cytotoxicity of hydrogels was evaluated using the directed contact 3-(4,5-dimethyl-2-thiazolyl)-2,5-diphenyltetrazolium bromide (MTT, Sigma-Aldrich, St. Louis, MO, USA) method, as previously described by our group [38]. Cells were cultured and tested in Dulbecco’s Modified Eagle Medium (DMEM, pH 7.4 ± 0.2, Sigma-Aldrich, St. Louis, MO, USA) with the addition of 10% fetal bovine serum (FBS, Cripion, São Paulo, SP, Brazil) as a supplement. The cells were incubated in contact with hydrogel samples for 24 h/37 °C. Control samples were designed as follows: control was cell culture with DMEM and 10% FBS; positive control (+Control) was cell culture with DMEM, 10% FBS, and 1.0% *v*/*v* Triton™ X-100 (Sigma-Aldrich, St. Louis, MO, USA); and negative control (−Control) was cell culture with DMEM, 10% FBS, and chips of sterile polypropylene Eppendorf^®^, 1 mg/mL (Eppendorf, Germany). The percentage of cell viability was used to estimate the cytotoxicity according to Equation (3). The values of the Control were considered 100% of cell viability response. The results were presented as the average ± standard deviation (n ≥ 4).

Cell viability (%) = (Absorbance of the sample and cells/Absorbance of control) × 100%(3)

#### 2.5.2. Hemocompatibility

The hemolytic index (HI) was applied to assess the hemocompatibility of the samples, determined according to the methodology proposed by Ghorpade et al. [43] with modifications. Hydrogels with 1.0 cm × 1.0 cm were allowed to swell in the phosphate buffer saline at 37 °C/1 h (n = 3). After that, PBS was removed, and 250 μL of mice blood (isogenic male BALB/c adult mice, Ethical Committee Approved Project Protocol No. 22/2018) with heparin (4 UI/mL) was added. The systems were left undisturbed for 20 min, and then, the hemolysis process was stopped by adding 1.75 mL of 0.9% NaCl saline solution. These samples were incubated (at 37 °C/1 h) and were centrifuged (at 4000 rpm/10 min), where the absorbance of the clear supernatant was measured at λ = 545 nm (spectrophotometer, Spectramax M, Molecular Devices). The hemolytic index values were calculated according to Equation (4). The results were presented as the average ± standard deviation (n = 3).

HI (%) = [(A_(sample)_ − A _(−control)_/(A_(+control)_ − A_(−control)_] × 100% (4)

where A = absorbance, +control = mixture of 250 μL of blood and 1.75 mL double-distilled water, and −control = mixture of 250 μL of blood and 1.75 mL of 0.9% NaCl saline solution.

### 2.6. In Vivo Biocompatibility Test—Mice Model

Balb/c animals weighing 20–25 g were obtained from the Bioterium Center of the Universidade Federal de Minas Gerais (CEBIO/UFMG), Brazil. These animals were kept in a room with a light/dark cycle of 12/12 h and free access to water and food. The Animal Ethics Committee approved the trial (CEUA—UFMG, protocol number 22/2018).

The animals received antibiotic cephalexin 15% (7.5 mg/kg, i.m.) 1 h before inserting the membranes (average diameter = 6.0 mm) in the subcutaneous region of the dorse. Three groups were evaluated at two different times (48 h and 28 days) after the procedure: (i) CTn—negative control (n = 4/time); (ii) CTp—positive control with murine collagen membrane (n = 5/time); and (iii) TR1—treatment with crosslinked hybrid hydrogel CMC/PVA/CA (n = 5/time).

The Balb/c animals were anesthetized with xylazine and ketamine (10 and 80 mg/kg, i.p., respectively), and the surgical site was submitted to asepsis with iodized alcohol after the trichotomy.

The skin was cut with scissors, followed by skin divulsion to the interscapular region, where the membranes were positioned. The same procedure was performed in the negative control animals; however, no membrane was inserted in the region after skin divulsion. Following the procedure of biomaterial insertion, the animal’s skin was sutured, and they were placed in boxes for recovery.

Finished the experimental periods (48 h and 28 days), the animals were euthanized by anesthetic overdose (i.e., ketamine 240 mg/kg and xylazine 30 mg/kg, i.p.), and the dorse skin was dissected at the site where the membranes were inserted and fixed in 10% buffered neutral formalin for 72 h.

Once fixed, the samples were submitted to dehydration in baths with increasing ethanol concentrations, diaphanized in xylol, and included in paraffin. Subsequently, a microtomy was performed, with sections 5 μm thick. The slides produced were stained by the hematoxylin-eosin technique for histopathological evaluation of the deep layer of the dermis, also called the adventitia layer, a region in close contact with the membranes tested.

### 2.7. Antibacterial Studies

#### 2.7.1. Agar Disk Diffusion Method

The effects of silver nanoparticles in the hydrogels and standard antimicrobials were tested against Gram-positive and Gram-negative bacterial strains using the disk diffusion method on Mueller–Hinton agar plates according to reported literature, i.e., the Clinical and Laboratory Standards Institute (CLSI) VET01, 5th edition [44], and M100, 28th edition [45]. Standard antimicrobial agents tested were norfloxacin and cefotaxime (Sigma-Aldrich, St. Louis, MO, USA).

All antimicrobials (Ag-functionalized hydrogels and standard antibiotics) were evaluated with the reference strains: *Pseudomonas aeruginosa* (Gram-negative, ATCC 27853), *Escherichia coli* (Gram-negative, ATCC 25922), *Enterococcus faecalis* (Gram-positive, ATCC 29212), and *Staphylococcus aureus* (Gram-positive, ATCC 29213). Briefly, all bacteria were grown on Mueller–Hinton agar plates (Acumedia Manufacturers Inc, USA) at 37 °C for 18 h in aerophilic conditions. After incubation, strains were suspended in a sterile 0.85% NaCl saline solution. The density was adjusted to 0.5 of the McFarland turbidity scale, corresponding to approximately 1.5 × 10^8^ CFU/mL (colony-forming unit), and applied to the Mueller–Hinton agar plates. Then, sterilized hydrogel disks at different Ag concentrations (average diameter = 6.0 mm; 30 min of UV irradiation on each side) and standard antibiotics (10 μg of norfloxacin and 30 μg cefotaxime in sterile filter disks) were added to the surface of inoculated plates. After incubating with samples at 37 °C for 18 h, inhibition zones were measured in millimeters. All assays were conducted in class II A2 biological safety cabinets in a sterile environment. Standard antimicrobials, norfloxacin and cefotaxime, and test protocols [44,45] were used to validate the results (positive controls), and media without bacterial inoculum were used as the negative control. Antibacterial assays were performed in triplicates (n = 3). The results were presented as average ± standard error.

#### 2.7.2. Bacteria Growth Inhibition Assay

The antimicrobial activities against *Escherichia coli* (ATCC 25922) and *Staphylococcus aureus* (ATCC 29213) were investigated using a growth inhibition assay followed by a colony-forming unit plate count as previously described in the literature with modifications [39,40,41]. The two bacterial strain suspensions were prepared as described in the previous Section 2.7.1, and the suspensions were serially diluted to obtain 5 × 10^6^ CFU/mL microbial concentration. Then, nanosilver-modified hydrogel membranes (CMC/PVA/CA_Ag015, CMC/PVA/CA_Ag030, and CMC/PVA/CA_Ag060) were incubated with 900 μL of Mueller–Hinton broth and 100 μL of 5 × 10^6^ CFU/mL suspensions for 4 h or 24 h at 37 °C. Previously to the experiment, the hydrogel membranes were cut into disks (average diameter = 5.5 mm) and sterilized (30 min of UV irradiation on each side), and one or three disks of each system were added to each vial for the assay. As controls, one tube with only culture media (negative control) and two tubes with bacterial inoculum in culture medium without hydrogel (positive control) were also evaluated.

At the end of each tested incubation period (4 h and 24 h), an aliquot of 100 mL of bacterial suspensions was collected, serially 10-fold diluted, and inoculated onto Mueller–Hinton agar plates, which were incubated at 37 °C for 18 h in aerophilic condition for bacteria count confirmation by standard technique. The detection limit was considered 10^2^ CFU/mL. All assays were conducted in class II A2 biological safety cabinets in a sterile environment. Two independent experiments with two replicates each were performed. The results were presented as average ± standard deviation.

The bacterial growth related to positive control (no disk) at a time “*t*” was calculated according to Equation (5) adapted from Xie et al. [46].

Bacterial Growth (*t*) (%) = [log(bacterial growth of sample (*t*)/log(bacterial growth of control (*t*)] × 100% (5)

### 2.8. Statistical Analysis

This study used Prism software (GraphPad Software, Version Prism 8, San Diego, CA, USA) for data analysis. Statistical significance was tested using one-way ANOVA followed by Bonferroni’s method. A value of *p* < 0.05 was considered statistically significant.

## 3. Results and Discussion

### 3.1. Characterization of Pristine Hybrid Hydrogel

#### 3.1.1. Physicochemical and Morphological Characterization of Pristine Hybrid Hydrogel

**Figure 1:**
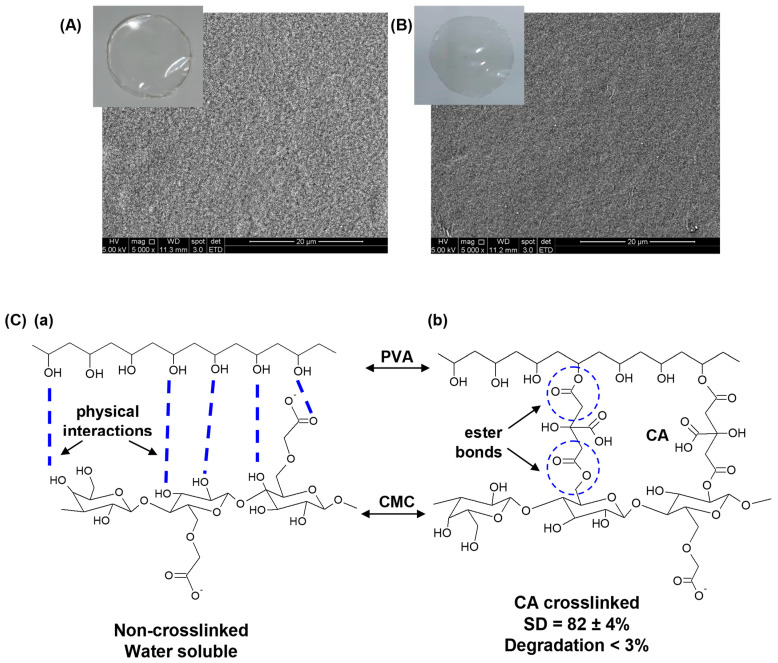
Digital images (insert, top left) and SEM images (5000×, scale bar = 20 μm) of (**A**) CMC/PVA (non-crosslinked) and (**B**) CMC/PVA/CA hydrogel. (**C**) Schematic representation of (**a**) physical interactions between polymers and (**b**) citric acid chemically crosslinked hybrid hydrogel (the drawings were presented with PVA and CMC, but these interactions were also expected to occur for PVA/PVA and CMC/CMC polymer chains).

The physicochemical and morphological characterization of pristine hydrogel was needed to compare with the hybrid systems formed after embedding silver nanoparticles in the matrices. Thus, a qualitative visual analysis was conducted to assess the most relevant macroscopic features of the CMC/PVA/CA hybrid hydrogels before and after chemical crosslinking with citric acid. They formed optically transparent and uniform membranes without evidence of phase separation or segregation (Figure 1A). At the microscopic level (Figure 1B), the non-crosslinked hydrogels exhibited a coarser morphology with granular features that became refined by chemical crosslinking, which is consistent with changes in the structural organization of polymeric chains forming the network. To be applied as wound dressing, hydrogels should present good chemical stability in an aqueous medium while they should swell, absorbing the excess fluid in the wound site. Thus, the non-crosslinked sample was unsuitable as it was completely water-soluble (CMC/PVA), relying mostly on weak and reversible interactions between the polymers due to physical crosslinking. Conversely, the hybrid hydrogel crosslinked with CA (CMC/PVA/CA) showed good stability, with 82 ± 4% water absorption (SD) and low degradation-solvation (100-GF) of less than 3% (24 h/DI water). These values are consistent with the esterification reaction between alcohols and carboxylic acids of polymers mediated by citric acid (Figure 1C). It reduces the number of hydrophilic hydroxyl groups while forming covalent bonds, rendering chemically crosslinked network structures with water stability.

**Figure 2:**
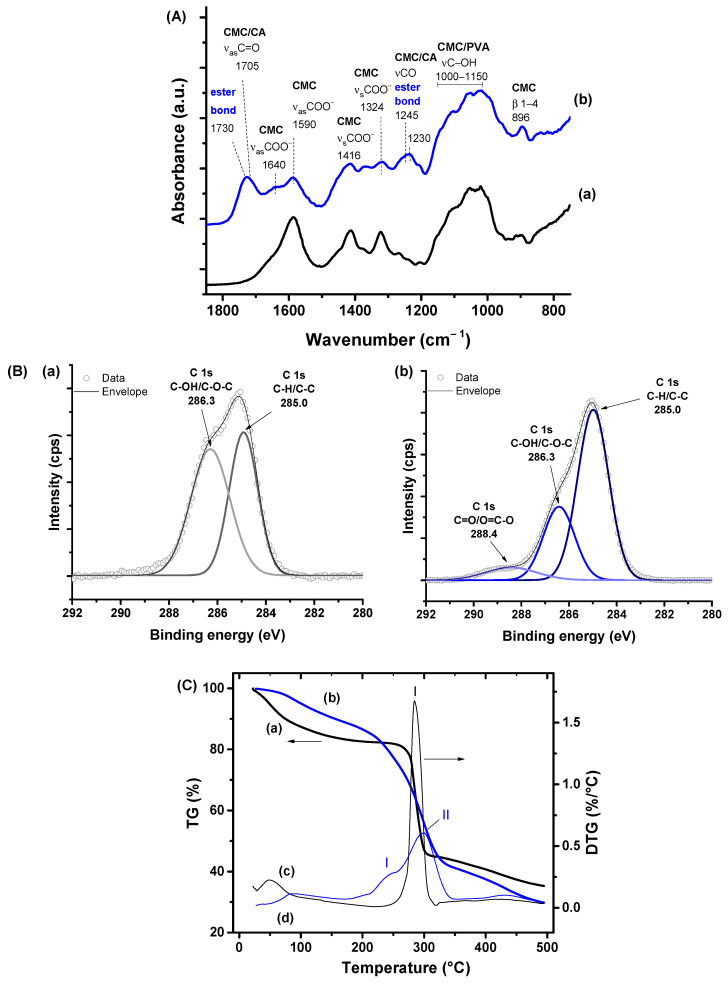
(**A**) FTIR spectra (range 1850–750 cm^−1^) and (**B**) XPS analysis of C 1s region obtained for (**a**) CMC/PVA and (**b**) CMC/PVA/CA. (**C**) (a,b) TG and (c,d) DTG analysis of CMC/PVA (a,c) and CMC/PVA/CA (b,d) hydrogels.

FTIR and XPS spectroscopy analyses were used to investigate the crosslinking reactions of CMC and PVA by CA to form the hydrogel matrices. As expected, the FTIR spectra (Figure 2A) of the hybrids of CMC and PVA were mostly dominated by the bands of CMC, the main component of the hydrogel (CMC: PVA 4:1 mass%). The major CMC and PVA vibrational bands before crosslinking are specified in Figure 2A(a). Upon the chemical crosslinking reaction (Figure 2A(b)), it was detected in the regions at 1730–1700 cm^−1^ and 1250–1230 cm^−1^ the vibrations related to ester bonds (R_1_-COO-R_2_) that were formed and CA incorporated to the hydrogel network. They validated the most accepted mechanism of crosslinking credited to the esterification reaction between carboxylic groups of the crosslinker (CA) with hydroxyl groups (-OH) of CMC and PVA polymers [10,23].

Regarding XPS analysis of the hydrogels, the results also showed important features that revealed the occurrence of crosslinking between polymer chains mediated by citric acid (Figure 2B). In the absence of the crosslinking agent (i.e., no CA, CA0, Figure 2B(a)), the hydrogels revealed the main characteristic peaks of CMC and PVA: 285.0 (C–H/C–C) and 286.3 (C–OH/C–O–C) [10,47]. Upon the addition of 25% CA and after the crosslinking reaction occurred, the XPS spectrum indicated a reduction in the relative peak area associated with the C–OH/C–O–C bond and, concurrently, a relative increase of the band associated with C–H/C–C binding energy. Endorsing the FTIR findings, the XPS result was ascribed to the consumption of the hydroxyl groups by the esterification reactions taking place, forming the polymeric hydrogel network. Moreover, a band overlapping the contributions of carbonyl (C=O) and esters and acids groups (O=C–O) at 288.4 eV, associated with CA and ester bonds, appeared compared to hydrogel without crosslinking [10,47].

Thermal analysis is valuable to support the findings discussed in the previous section regarding the stability and the polymer hybrid network formation. Thus, the thermal profiles of hydrogels were also affected by the crosslinking reaction (Figure 2C). For both CMC/PVA hydrogels, before and after crosslinking (CA0 and CA25), two main stages of mass loss were observed up to 500 °C. The first is related to the mass loss mostly associated with water removal (moisture and adsorbed water) up to 200 °C, followed by polymer degradation at the 2nd stage. The mass loss associated with degradation occurs from 250 °C in a single event for uncrosslinked samples. In contrast, for the crosslinked samples (CA25), it was observed at 180 °C, overlapping two thermal events, representing a reduction in the thermal stability of hydrogels. This effect could be associated with changes in the overall organization of polymer chains as, upon crosslinking, the intra- and intermolecular bonds between functional groups (physical interactions) were partially replaced by ester bonds (chemical crosslinking), leading to a lower degradation temperature of hydrogels [10].

Furthermore, bearing in mind the main goal of applying these hybrids for wound dressings and temporary skin tissue substitutes, the properties of permeability, wettability/hydrophilicity, and surface charges are important. They would maintain a balanced moisture microenvironment at the wound site, avoiding undesired exudate excess or dryness and influencing the interactions with cells and tissues. The permeability of CMC/PVA/CA evaluated by water vapor transmission (WVT) was 338 ± 5 g.m^−2^.d^−1^. This value is compatible with the wide range reported for commercial wound dressings (76–9360 g.m^−2^.d^−1^) and healthy skin tissues (~200 g.m^−2^.d^−1^) [10,48]. Additionally, the wettability and the surface charge of CMC/PVA/CA hydrogels were assessed by the contact angle (θ) and point of zero charge (PZC), respectively. The contact angle (θ) was 55 ± 1° and PZC was 4.9 ± 1.

The results of both parameters indicated a moderate hydrophilic surface and the occurrence of positive charges at physiological conditions, which were expected to be more appropriate for promoting cell attachment, adhesion, and proliferation [11,49,50].

#### 3.1.2. Biological Tests

##### Cytocompatibility

**Figure 3:**
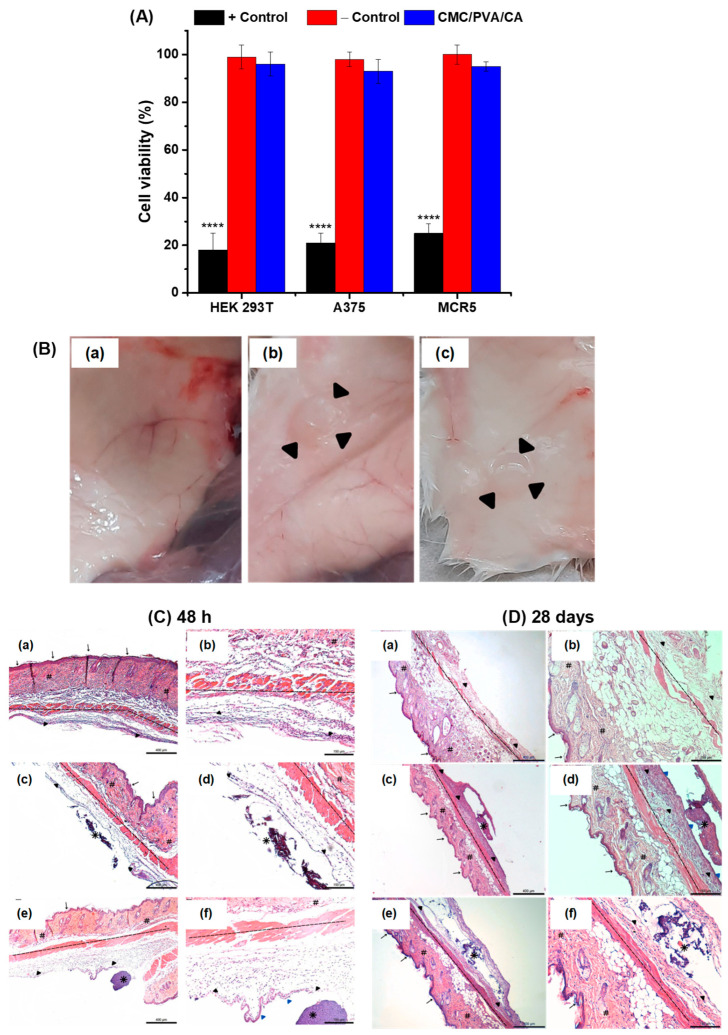
(**A**) Cell viability results of HEK 293T, A375, and MCR5 cell cultures based on MTT protocols after 24 h of incubation with CMC/PVA/CA hybrid hydrogel. (**B**) Demonstrates the absence or presence of features resembling the presence of the membrane (arrowheads) four weeks after the surgical procedure. (**a**) CTn; (**b**) CTp; (**c**) TR1. Histological images of the epithelial tissue in the groups after (**C**) 48 h and (**D**) 28 days, in a smaller 4× magnification (**a**: CTn; **c**: CTp; **e**: TR1, scale bar = 400 μm) and in greater increase 40× (**b**: CTn; **d**: CTp; **f**: TR1, scale bar = 150 μm). The arrows indicate the region of the epidermis; the hashtag indicates the dermis; the dashed line indicates the muscle tissue; the black arrowheads show the region of the adventitia, the blue arrowheads show the epithelialized region of the tissue, and asterisks the membranes (CTp and TR1) content (‘one way’/ANOVA/Bonferroni. Significant differences compared with “−Control”; *p* < 0.0001 = ****).

The cytotoxicity of the CMC/PVA/CA hydrogels was evaluated using the directed contact 3-(4,5-dimethyl-2-thiazolyl)-2,5-diphenyltetrazolium bromide (MTT) method. The CMC/PVA/CA hydrogels exhibited elevated cytocompatibility with cell viability responses higher than 90% after being incubated for 24 h (Figure 3A), with 3 distinct cell cultures: (a) MCR5, fibroblasts isolated from the lung tissue; (b) A375, human malignant melanoma cells; and (c) HEK 293T, human embryonic kidney cells. These cytocompatibility findings are very important as, beyond physicochemical properties and morphological features discussed in previous sections, they confirmed the favorable biomedical application of these chemically crosslinked polymer-based hybrids as wound dressings.

##### Hemocompatibility

As a vital complementary in vitro assay for assessing biocompatibility, the hemocompatibility response of the pristine polymer-based hydrogel was evaluated through hemolysis tests. The hemolysis index (HI) signifies the extent of red blood cells broken by the sample in contact with whole blood. As a reference [11,51], HI values must be lower than 2.0% to classify the material as non-hemolytic. Hence, the average HI value for the CMC/PVA/CA hydrogel hybrids was 1.1 ± 0.1%, similar to those obtained for collagen, considered the reference standard biomaterial (HI = 1.1%). In this sense, the hydrogels showed hemocompatibility and were considered non-hemolytic. At this point, it could be summarized that the designed polymer-based hydrogels, composed of CMC-PVA-CA, were effectively produced and extensively characterized in vitro, demonstrating to fulfill the crucial requirements for qualifying as a wound dressing and potentially assisting chronic wound healing.

##### In Vivo Biocompatibility

Hence, to move beyond the in vitro cytocompatibility and hemocompatibility tests, in vivo bioassays were also performed in murine animal models. To observe the biocompatibility of the CMC/PVA/CA hydrogel hybrid membranes in the murine tegument, they were evaluated regarding the possibility of promoting changes in the subcutaneous region of the dorse after insertion at two time intervals, 48 h (2 days) and 28 days. Moreover, when considering the mechanical characteristics for potential wound healing applications, these hydrogels were revealed to be soft, flexible, adaptable in shape, moisture-absorbent, physically and chemically stable to be positioned in the wound site, filling the cavity or lesion. Besides that, these hydrogels were also demonstrated to be easily handled and properly well fitted in the wound site, confirmed via this in vivo mice model.

In the first stage, macroscopically, these changes were observed only after 28 days (Figure 3B) when the animals of the CTp (positive control, Figure 3B(b)) and TR1 (treatment with CMC/PVA/CA, Figure 3B(c)) groups showed the formation of small pockets in the membrane fixation region. In the second stage, the tegument regions were microscopically analyzed (Figure 3C,D) at the respective times and compared with the CTn group (negative control). After 48 h, it was possible to observe the presence of inflammatory infiltration in all groups, being more evident in the CTn group (Figure 3C(a,b), more evident edema, and possible epithelium development in TR1 (Figure 3C(e,f). Regarding the time of 28 days, it was observed that the murine collagen membrane, used as the standard skin control, stimulated the formation of a capsular structure consisting of fibrous connective tissue with discrete inflammatory infiltrate. The adventitia layer presented mild edema and the formation of an area of epithelialization adjacent to the inserted material (Figure 3D(c,d). Using the CMC/PVA/CA hydrogel increased the inflammatory infiltration, edema (moderate), and blood vessels in the adventitia (Figure 3D(e,f). These qualitative results demonstrated that the insertion of hybrid hydrogel membranes in the dorse subcutaneous region of mice may have caused changes such as a moderate increase of inflammatory infiltrate, mild edema, and the formation of a fibrous capsule neighboring the tested materials. These in vivo results relying on mice animal models are well matched with other reported studies [52], demonstrating that the CMC/PVA/CA hydrogels (TR1) were biocompatible, endorsing the in vitro biological assays discussed in the previous sections. Consequently, the combination of results of this research validates that, from a physicochemical and biological strategy (i.e., in vitro and in vivo), these innovative hydrogel membranes of CMC/PVA/CA could be potentially suitable for wound dressing and skin repair substitutes. Nonetheless, future in vivo experiments using diabetic-induced mouse models will be needed to further validate the application of these hybrids in assisting the wound healing process.

### 3.2. Characterization of Nanosilver and Ag-Functionalized Hybrid Hydrogels

One of the most critical aspects related to diabetic chronic wounds is associated with the high rates of microbial infections, which drastically increase medical complications. Thus, silver nanoparticles (AgNPs) were synthesized to address this important point, focusing on creating hydrogel polymeric matrices with antibacterial activity (Ag-functionalized hybrid hydrogels). AgNPs were prepared using an aqueous eco-friendly process based on the mixture of CMC/PVA polymer solutions, which functional groups (predominantly hydroxyls) adsorbed and acted as chemical reductants for nucleating silver nanoparticles in situ (i.e., reduction: Ag^+^ → Ag^0^; R-OH oxidation) while stabilizing the colloidal dispersion as capping ligands [37,53]. Different concentrations of silver nanoparticles in colloidal suspensions were achieved by changing the concentrations of silver salt precursor, keeping the concentrations of CMC and PVA constants, which was required to investigate the antimicrobial activity while not provoking cytotoxicity to the skin cells. Then, AgNP-functionalized hybrid hydrogels were obtained upon adding citric acid as the crosslinker to the nanosilver colloidal solution, followed by the thermal treatment described in the Materials and Methods section.

#### 3.2.1. Characterization of Nanosilver

**Figure 4:**
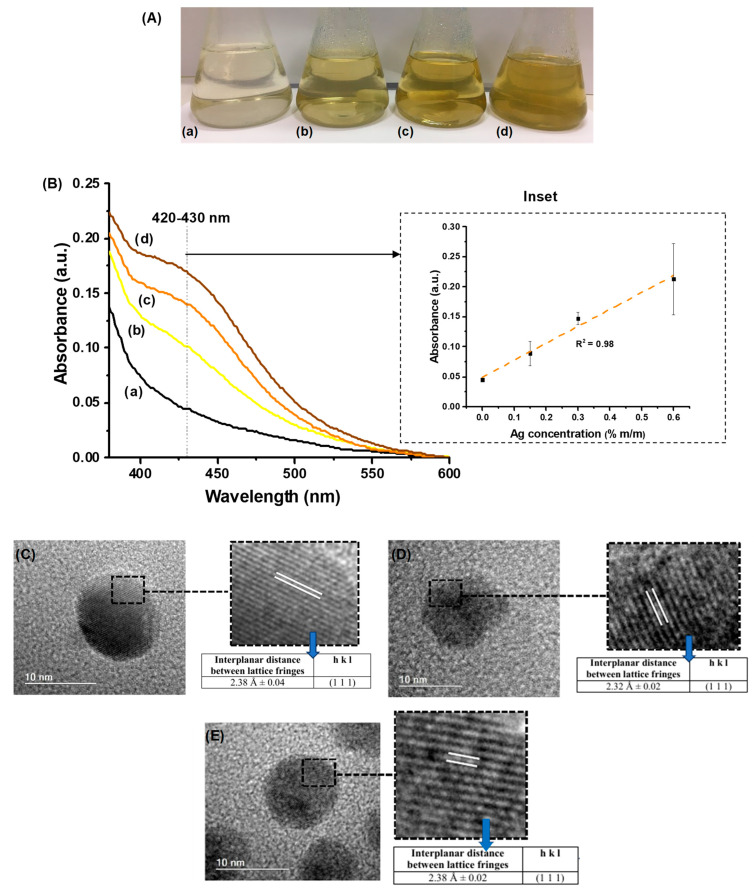
(**A**) Digital images of colloidal dispersions and colors changing to a light yellowish-orange color solution accordingly with the colloidal dispersion silver concentration degree ((**a**) Ag0, (**b**) Ag015, (**c**) Ag030, and (**d**) Ag060). (**B**) UV–vis spectra of CMC-PVA_Ag dispersions ((a) Ag0, (b) Ag015, (c) Ag030, and (d) Ag060). Inset: UV absorbance intensity at 430 nm at different Ag concentrations. HRTEM images with interplanar distance between lattice fringes (inset) obtained from colloidal suspensions: (**C**) Ag015; (**D**) Ag030; (**E**) Ag060.

The silver cations were chemically reduced to metallic particles by in situ reaction, which were qualitatively observed by the visual changes from colorless to the light yellowish-orange color of polymer solutions, gradually increasing with the evolution of the reaction. As expected, it was also noticed that the relative intensity of color was increased following the nanosilver content in the dispersions, from Ag0 (without AgNPs) to Ag60 (0.60 m/m% [Ag]/[CMC + PVA]) (Figure 4A).

In addition, the formation and stabilization in situ of AgNPs using CMC/PVA in water solution were assessed by UV–vis spectroscopy of colloidal dispersions (Figure 4B). It could be observed a band of absorption at approximately λ = 420–430 nm, despite the Ag content of samples (Figure 4B(b–d)), in comparison to CMC/PVA solution without AgNPs (Figure 4B(a)). These results were assigned to the surface plasmon resonance (SPR) associated with forming metallic Ag particles at the nanoscale dimensions [37,54]. Based on the literature, the SPR phenomenon is generated by the incident electromagnetic wave interacting with metal nanoparticles. As a consequence, SPR leads to absorption and scattering of the incident electromagnetic wave, shown by the emergence of an intense band in the UV–vis spectrum [55,56]. It could also be observed that the increase in the amount of silver nitrate enhanced the intensity of the absorption peak (inset in Figure 4B), which indicates a higher concentration of silver nanoparticles. From a statistical and physicochemical approach, the value of R^2^ = 0.98 (inset in Figure 4B) quantitatively indicated that the data well matched the linear regression model in our study. That shows that the in situ reduction of silver cations (Ag^+^) in an aqueous solution by polymer functional groups (i.e., hydroxyls) correlated fairly well with the absorbance measurements credited to nanosized silver metallic particles formed in a colloidal solution due to the SPR phenomenon. Additionally, a more in-depth investigation of the mechanism of AgNP reduction is beyond the scope of this research, and it is well established in the literature [37].

The “as-synthesized” AgNPs dispersed in aqueous colloidal media were evaluated by TEM image analysis, as presented in Figure 4C–E. As a general trend, it can be observed that CMC/PVA silver nanoparticles reduction/stabilization produced similar nanoparticles with spherical morphology and sizes ranging from 2–12 nm. In addition, the crystallinity of AgNPs was further investigated by HRTEM (high-resolution-transmission electron microscopy) imaging. The presence of lattice fringes confirmed the crystallinity of the nanoparticles. HRTEM images of the CMC/PVA_Ag015 (Figure 4C), CMC/PVA_Ag030 (Figure 4D), and CMC/PVA_Ag060 (Figure 4E) indicated an interplanar distances d = 2.38 ± 0.04 Å, d = 2.32 ± 0.02 Å and d = 2.38 ± 0.02, respectively, consistent with the (1 1 1) plane of the metallic silver (JCPDS—87-0720 and JCPDS—04-0783). Hence, these results demonstrated the formation of crystalline metallic AgNPs, which were effectively produced by the in situ chemical reduction of Ag^+^ in CMC/PVA aqueous media (i.e., Ag^+^/CMC-PVA → Ag^0^/CMC-PVA colloid), supported by the literature [37,57].

#### 3.2.2. Characterization of Nanosilver-Functionalized Hybrid Hydrogels (CMC/PVA/CA_AgNP)

**Figure 5:**
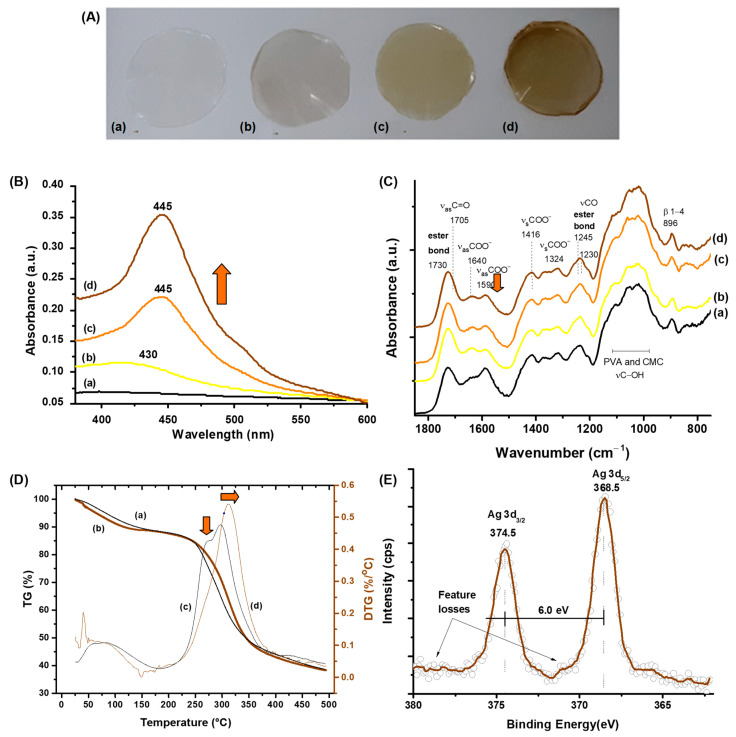
Nanosilver-functionalized hybrids: (**A**) digital photos, (**B**) UV–vis spectra, and (**C**) FTIR spectra of pristine CMC/PVA/CA membrane (a, Ag0) compared to CMC/PVA/CA_Ag membranes with (b) Ag015, (c) Ag030, and (d) Ag060; arrow for carboxylate (COO^−^) at 1590 cm^−1^. (**D**) TG (a,b) and DTG (c,d) curves of pristine (a,c) and Ag-functionalized hydrogel (b,d, CMC/PVA/CA_Ag060). (**E**) Typical high-resolution XPS spectrum of Ag 3d region of hybrid hydrogels (CMC/PVA/CA_Ag060).

To support potential applications in diabetic chronic wound healing, further characterizations were required to confirm that key properties and morphological features of pristine synthesized hydrogel membranes were retained after incorporating the silver nanoparticles forming the hybrid polymeric matrices. Three concentrations of AgNPs (0.15, 0.30, and 0.60 m%) embedded in CMC/PVA polymer matrices and crosslinked by citric acid (CA/[CMC + PVA] = 25%) were investigated. Typical hybrid membrane images are shown in Figure 5A. Visually, a color change of the membrane was observed from colorless (Ag0), semitransparent light yellowish color (Ag015), and with a gradual increase to dark orange for the Ag030 and Ag060 samples, following the degree of silver nanoparticle content, a similar trend of the colloidal suspensions. The hybrids incorporated with nanosilver were uniform and homogenous without any noticeable phase segregation, and the average thickness of the hydrogel membranes was 45 ± 1 μm, compatible with superficial cutaneous wounds.

When comparing the swelling degree values of CMC/PVA/CA_Ag, it can be observed that they tend to be similar regardless of the AgNP concentration embedded into the hydrogels with SD = 73 ± 2%. This result showed no significant statistical variation (‘One way’/ANOVA/Bonferroni, α = 0.05) regarding pristine hydrogel (without AgNP, SD = 82 ± 4%, Section 3.1.1) by incorporating the silver nanoparticles into the hybrid matrices. An analogous trend was observed for degradation property. The results demonstrated no significant difference between CMC/PVA/CA_Ag nanocomposite membranes and pristine hydrogel (‘one way’/ANOVA/Bonferroni, α = 0.05) with degradation (100-GF) of approximately 2 ± 1% for both systems. These results showed that incorporating AgNPs did not affect these two important properties of hydrogels crucial for wound dressing applications. It demonstrated that the chemical stability of the hydrogel required for the healing process was preserved while offering a balanced moisture absorption microenvironment assisting the wound healing process [10,37].

As previously discussed in the colloidal synthesis of AgNPs (Section 3.2.1), the UV–vis results (Figure 5B) indicated the typical absorption spectra associated with surface plasmon peaks (SPR) with maxima at ca. 430–445 nm. This SPR range is compatible with nanosized silver particles embedded in the hybrid polymer matrices, which can be affected by several aspects, including morphology, size, surface charges, and dielectric features of the matrix. As a general trend, as expected, increasing the AgNP concentration from 0.15% to 0.60% led to higher absorbance of the hybrid membrane (Figure 5B, arrow). Although the incorporation of silver nanoparticles was projected chiefly to assign antibacterial properties to the hybrids, the polymer-based hydrogel matrices were extensively characterized regarding their physicochemical and structural properties where no major changes were detected. Thus, these results confirmed their suitability as wound dressings tailored to assist diabetic chronic wound healing.

Regarding FTIR spectroscopy (Figure 5C), changes in the absorbance intensities of carboxylate (COO^−^) asymmetric stretching (1589 cm^−1^ and 1637 cm^−1^) bands were observed in the spectra of Ag-functionalized hydrogels (Figure 5C(b–d) in comparison to hydrogel without silver nanoparticles (Figure 5C(a), Ag0), without shift of their wavenumbers. This aspect could be associated with the interaction of Ag species with the negatively charged carboxylate groups of CMC. Moreover, no substantial changes were noticed in the vibrations of ester bonds formed in the crosslinking reactions of polymer chains (CMC and PVA) mediated by citric acid in the presence of silver nanoparticles.

Likewise, the thermal behavior of hydrogels was not significantly affected by the presence of AgNPs (Figure 5D). The Ag-functionalized samples followed the same thermal profile as pristine samples with two main stages accompanied by similar mass loss with degradation of polymer chains starting at approximately 180 °C. The only change detected was the reduction of the mass loss at the first event of degradation balanced by an enhancement of the loss in the second event, in which the maximum was shifted to higher temperatures with increasing Ag content (Figure 5D, arrows).

The effect of chemical reduction of Ag^+^ species forming metallic colloidal Ag (CMCPVA_AgNPs) and their surface features after crosslinking reactions were assessed by XPS spectroscopy (Figure 5E). Typical XPS spectra of nanosilver-functionalized hydrogels presented peaks at 374.5 ± 0.2 eV and 368. ± 0.2 eV, Ag 3d_3/2_, and Ag 3d_5/2_, respectively, with the spin-orbit components split by a binding energy interval of 6.0 eV. These aspects, combined with loss features at the higher binding energy side of the spin-orbit components, endorsed the in situ formation of metallic silver by redox chemical reactions with polymers [37,58]. Moreover, these results validate the effective presence of metallic silver nanoparticles, with no detectable oxidation at the surfaces, by functionalizing the polymer-based hydrogel membranes.

### 3.3. Characterization of Nanosilver-Functionalized Hybrid Hydrogels—Biological Assays

#### 3.3.1. Antibacterial Activity

##### Agar Disk Diffusion Method

Four reference strains were tested: two Gram-positive (*E. faecalis* and *S. aureus*) and two Gram-negative (*E. coli* and *P. aeruginosa*). *S. aureus*, *E. coli*, and *P. aeruginosa* are among the most common microorganisms found in diabetic foot ulcers [41,59].

**Figure 6:**
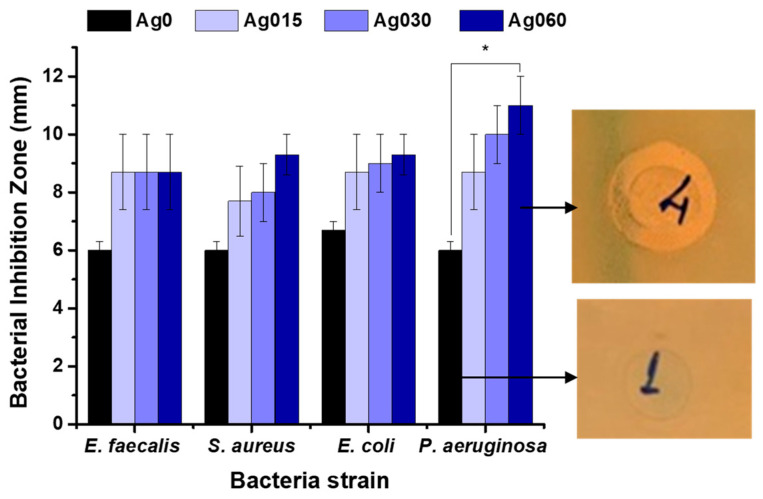
Representative disk diffusion test performed with different strains (*E. faecalis*, *S. aureus*, *E. coli*, and *P. aeruginosa*) to test the antimicrobial activity of nanosilver hybrid hydrogels (Ag0 = CMC/PVA/CA; Ag015 = CMC/PVA/CA_Ag015; Ag030 = CMC/PVA/CA_Ag030; Ag060 = CMC/PVA/CA_Ag060). Digital images of inhibition halos for *P. aeruginosa* in contact with reference hydrogel (no AgNPs) and CMC/PVA/CA_Ag060 (‘one way’/ANOVA/Bonferroni. Significant differences of Ag-functionalized hydrogels with Ag0; *p* < 0.05 = *).

For disk diffusion assay (Figure 6), Ag-functionalized nanocomposites demonstrated in vitro antibacterial activity compared with pristine (CMC/PVA/CA, no AgNPs) owing to the presence of embedded AgNPs. The diameter of the inhibition zone agrees with reports in the literature for nanocomposites enriched with AgNPs, as presented in the review of Kalantari et al., 2020 [60]. In addition, the nanosilver-functionalized hydrogels showed a trend of improving antibacterial activity from the lowest concentration of 0.15% AgNP to the highest concentration of 0.60% AgNP, even better against Gram-negative isolates, with CMC/PVA/CA_Ag060 for *P. aeruginosa* being significantly different from pristine hydrogel (Ag0).

*E. faecalis*, *S. aureus*, *E. coli*, and *P. aeruginosa* were used as assay quality standard control against two antimicrobials from different classes, norfloxacin (fluoroquinolone) and cefotaxime (cephalosporin). All controls showed inhibition halos (Table S1) compatible with the intervals described in the CLSI M100 reference manual [45], which validated the assays (Table S2).

##### Bacteria Growth Inhibition Assay

Two representative strains of *E. coli* and *S. aureus* were assayed using a growth inhibition procedure followed by a colony-forming unit (CFU) plate count to progress on investigating the antibacterial activity of the developed hybrid hydrogels. The tests were performed with the different nanocomposites (Ag0 = CMC/PVA/CA; Ag015 = CMC/PVA/CA_Ag015, Ag030 = CMC/PVA/CA_Ag030, and Ag060 = CMC/PVA/CA_Ag060) using one or three disks (for example, Ag015_1disk and Ag015_3 disks) of the samples to increase the amount of silver content for each system.

**Figure 7:**
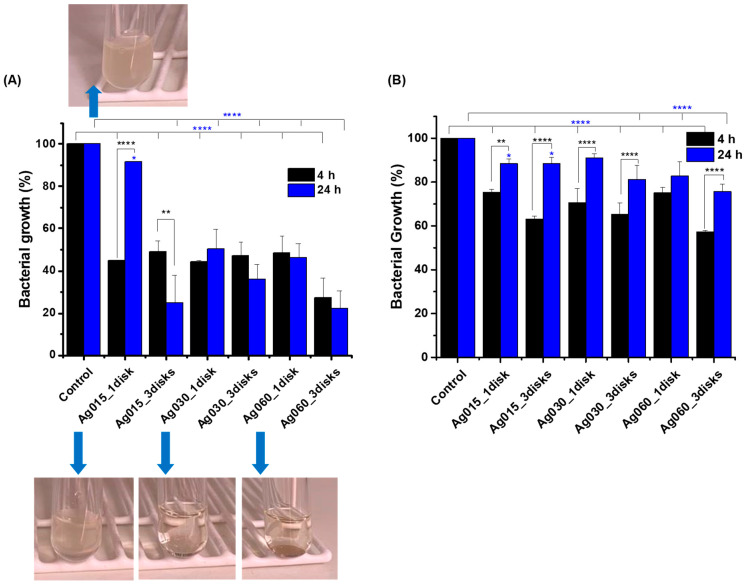
Bacterial growth inhibition of (**A**) *E. coli* and (**B**) *S. Aureus* after 4 h and 24 h treatment with Ag-functionalized hybrid membranes at different concentrations. Digital images of *E. coli* suspensions after 24 h of incubation with positive control (no disk) and 1 disk of different samples (‘one way’/ANOVA/Bonferroni. Significant differences compared with control (blue asterisks) and comparing 4 h and 24 h for the same system (black asterisks). *p* < 0.05 = *, *p* < 0.01 = **, *p* < 0.0001 = ****).

The bacterial inhibition of the nanosilver-modified hydrogels is presented in Figure 7. The results showed an inhibitory action of the nanosilver-modified hydrogels since the CFUs in all treatments were significantly lower compared to positive controls (no disk). Moreover, it is worth mentioning that the negative control also validated the study, as no growth was observed in the tubes containing only broth, which guarantees the sterility of the process, and that the bacterial growth observed was only of the bacteria of interest.

In addition, through comparisons between treatments and controls, it was possible to observe that, in some cases, but especially for *S. aureus*, there was a significantly greater antimicrobial action within the first 4 h of incubation, with an additional increase in CFU after 24 h. This activity can be interpreted by a rapid bactericidal action followed by a bacteriostatic phase. Over time, antimicrobial compounds become depleted, and microorganisms begin to grow again in situations where the bacteria initially present were not completely inhibited. In this sense, the clinical recommendation would be to re-administer the antimicrobial recurrently until all microorganisms are inhibited.

Also, as anticipated, a trend of greater growth inhibition was observed with the increased number of disks and tests with systems with higher silver concentrations for both microorganisms. The inhibition growth against *E. coli* was comparatively much higher than *S. aureus* for 4 h and 24 h. This behavior has also been observed in published studies using silver nanoparticles for similar purposes and treating oral microorganisms [41,61,62].

Although the antimicrobial mechanism of silver nanoparticles is not entirely elucidated yet, the main theories are associated with the ability to absorb silver species (metallic and ions) by bacterial cells. This process is favored considering that Gram-negative and Gram-positive bacteria have a negatively charged surface due to their polar lipid bilayers. Consequently, the release of silver ions by nanoparticles disrupts the bacterial cell membrane, leading to strong interactions with biomolecules such as proteins, membranes, and DNA. Ag ions and nanoparticles can also potentially stimulate the production of cytotoxic reactive oxygen species (ROS) inside the cells.

Moreover, since the cell wall of Gram-negative bacteria is relatively thinner than that of Gram-positive bacteria, this may favor the penetration of nanoparticles into the wall and justify the better action of silver on these strains. The literature supports these findings of a superior antibacterial action on *E. coli* than on other Gram-negative microorganisms [62,63,64,65].

#### 3.3.2. Cytotoxicity Characterization by MTT Assay

After being subject to antibacterial activity evaluation, the CMC/PVA-based hybrid membranes incorporating AgNPs were evaluated for cytocompatibility and compared with pristine hydrogels, considered the reference matrices. The preliminary assessment of cytocompatibility of the hydrogels before and after the incorporation of AgNPs was based on analysis of the cell viability of lung fibroblasts (MCR5), human embryonic kidney (HEK 293T), and human malignant melanoma cells (A375) using the in vitro MTT assay (direct contact method; samples of 16 mm^2^; Ag concentrations of 1.2 μg for CMC/PVA/CA_Ag015, 2.4 for CMC/PVA/CA_Ag030 μg, and 4.8 μg for CMC/PVA/CA_Ag060).

**Figure 8:**
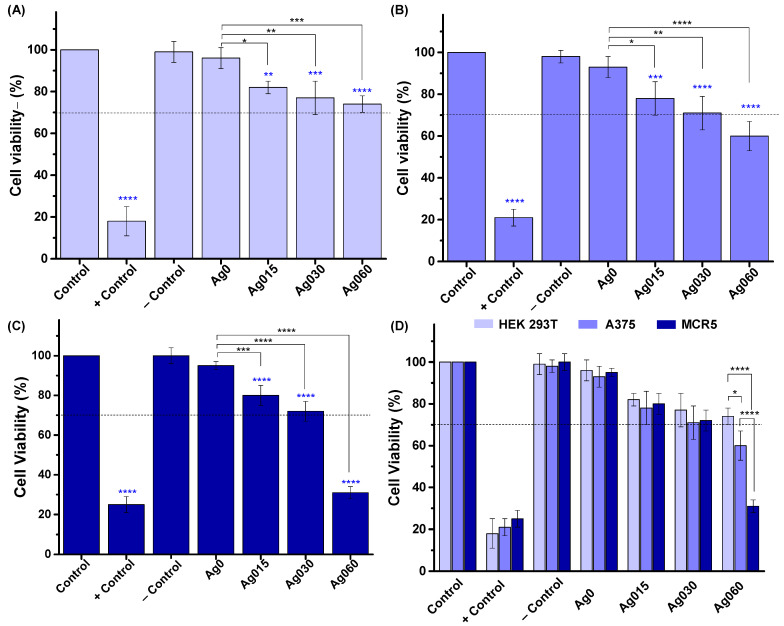
Cell viability response by MTT in vitro assay ((**A**) HEK 293T, (**B**) A375, and (**C**) MCR5, and (**D**) comparison of previous results) after 24 h of incubation with 16 mm^2^ Ag nanocomposites with different concentrations of Ag (Ag0 = CMC/PVA/CA; Ag015 = CMC/PVA/CA_Ag015; Ag030 = CMC/PVA/CA_Ag030; and Ag060 = CMC/PVA/CA_Ag060) (‘one way’/ANOVA/Bonferroni. (**A**–**C**) Significant difference compared to control (blue asterisks) and Ag-functionalized hydrogels with Ag0 (black asterisks). (**D**) Significant differences comparing samples of the same group of Ag for different cell lines. *p* < 0.05 = *, *p* < 0.01 = **, *p* < 0.001 = ***, *p* < 0.0001 = ****).

Based on the results in Figure 8, samples without being functionalized with nanosilver nanoparticles (CMC/PVA/CA) demonstrated cytocompatibility towards the three cell lines (statistically equal to control). This important result was predominantly attributed to the well-known high biocompatibility of CMC and PVA polymers producing the hydrogels, combined with the rational choice of citric acid as a “green and naturally sourced” crosslinker agent.

The effect of Ag nanoparticles incorporated in the hydrogel hybrid matrices was verified by the relative reduction of cell viability compared to CMC/PVA/CA hydrogels without AgNPs. As a general trend, a decrease in cell viability response was observed for higher contents of AgNPs (CMC/PVA/CA_Ag060). Nonetheless, these hybrid samples with Ag015 (1.2 μg) and Ag030 (2.4 μg) embedded in the membranes also showed cell viability higher than 70% and, therefore, are considered nontoxic. These findings indicate a suitable cell response according to the widely accepted international standard (10993-5:2009) applied for assessing cytocompatibility of biomaterials (Tests for in vitro cytotoxicity) [42]. As discussed in the topic regarding the antibacterial activity of AgNPs, the possible cytotoxicity of AgNPs has been mostly associated with the generation of oxidative species, DNA damage, and the release of intracellular Ag being dose-dependent, as observed in the results. Moreover, nanosized material favors the decrease of cell viability due to the stronger reactivity caused by a higher surface-to-volume ratio and increased cell uptake [37,66]. The results also indicated a significantly higher reduction of cell viability for the MCR5 cell line. According to the literature [37,66], the cell line can influence responses to AgNPs, with some cell lines being more resistant. In contrast, others are more sensitive to the effects of AgNPs as they can endure different stimuli in the types of cells. These findings are of pivotal importance as they confirmed the opposite tendency of antibacterial activity and cell viability when incorporating silver nanoparticles into hybrid hydrogels. It can be considered that AgNPs could eliminate pathogens like bacteria but can also provoke cytotoxicity in mammalian cells [37,66]. Thus, the concentration of AgNPs should be fine-tuned for producing polymer-based hydrogels with a “smart” balance between antibacterial activity and cell viability to assist in chronic wound healing applications.

**Figure 9:**
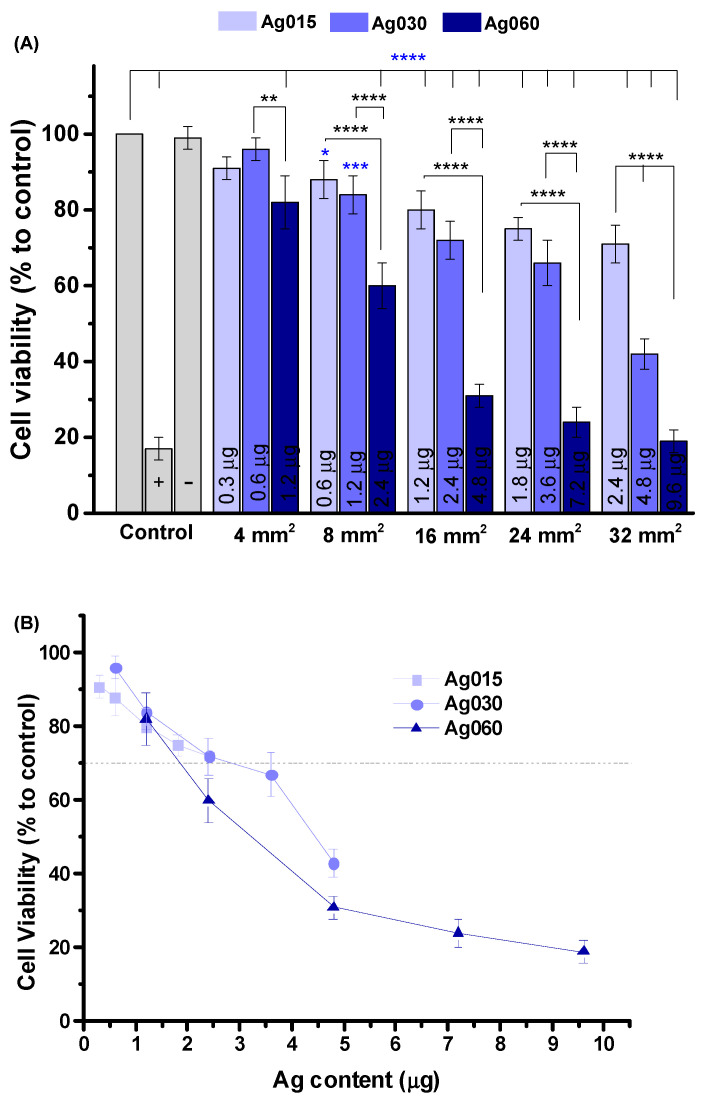
(**A**) Cell viability response of human pulmonary fibroblast cells (MRC5) culture based on MTT assay after 24 h incubation in contact with the developed membranes with different pad sizes (Ag0 = CMC/PVA/CA; Ag015 = CMC/PVA/CA_Ag015; Ag030 = CMC/PVA/CA_Ag030; and Ag060 = CMC/PVA/CA_Ag060). (**B**) Cell viability x Ag content (‘one way’/ANOVA/Bonferroni. Significant differences compared to control (blue asterisks) and among Ag-functionalized hydrogels (black asterisks). *p* < 0.05 = *, *p* < 0.01 = **, *p* < 0.001 = ***, *p* < 0.0001 = ****).

Figure 9A shows the MTT assay results of cell viability responses of the MCR5 cell line toward the hybrid membranes with different nanocomposite membrane sizes for the three Ag concentrations (which results in different content in the mass of Ag in the membranes, as indicated in the columns). As a general trend, the cell viability responses depended on the area of the hybrid membrane pad in contact with the cells and the relative concentration of silver nanoparticles. At a smaller pad area (4 mm^2^), despite minor differences with the reference (CMC/PVA/CA, no AgNPs), the CMC/PVA/CA_Ag015 and CMC/PVA/CA_Ag030 hydrogels demonstrated equivalent cytocompatibility towards MRC5 cells. At this membrane size, Ag060 nanocomposites were also non-cytotoxic despite being different from the reference sample. For larger hybrid Ag-functionalized membrane pad sizes, the cell viability responses gradually decreased with increasing the Ag content. However, CMC/PVA/CA_Ag060 hybrids significantly reduced cell viability response as membrane size increased. Thus, considering that the cytocompatibility of materials for biomedical applications must be superior to 70% [42], this hybrid at larger sizes is not recommended to be applied due to their measured reduced cell viability, indicating cytotoxicity. At the same time, all other hybrids with concentrations of AgNPs (i.e., 0.15% and 0.30%) with pad sizes smaller than 24 mm^2^ were qualified for testing as wound dressing, considering all properties investigated, including their cell viability > 70%.

Figure 9B summarizes the window of Ag concentration versus cell viability. As silver nanoparticles act like a *dual-functional nanomaterial*, they can eliminate bacteria but may induce cytotoxicity. These findings are very important considering the wound dressing application. The relative concentration of silver nanoparticles incorporated into the pad could be tuned for the individual patient condition, bearing in mind the extension of infection, bacteria strain, and wound site dimensions, among others, reducing harmful effects and using its benefit as an antibacterial agent.

#### 3.3.3. Hemocompatibility Study

**Figure 10:**
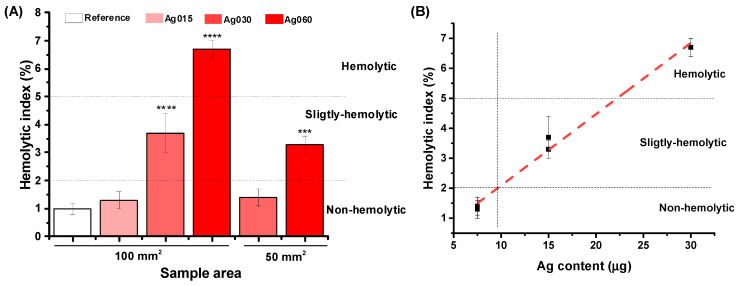
(**A**) Hemolytic index for 100 mm^2^ and 50 mm^2^ samples functionalized with AgNP (Ag015 = CMC/PVA/CA_Ag015; Ag030 = CMC/PVA/CA_Ag030; and Ag060 = CMC/PVA/CA_Ag060). (**B**) Dependence of HI on Ag content (‘one way’/ANOVA/Bonferroni. Significant differences compared to reference (collagen). *p* < 0.001 = ***, *p* < 0.0001 = ****).

Hemocompatibility is another key property when considering synthetic material suitability for wound dressing and skin repair. According to ASTM F 765-00 [67], materials are classified as non-hemolytic when 0 > HI > 2, slightly hemolytic when 2 > HI > 5, and hemolytic when HI > 5. Thus, like reference material (collagen, HI = 1.0 ± 0.2%), the hybrid membranes of 100 mm^2^ with 0.15% nanosilver particles were found to be non-hemolytic. For 0.30% of AgNP, it was slightly hemolytic [11,51]. However, the highest AgNP concentration of 0.60% displayed undesirable hemolytic activity (Figure 10A). Upon reduction of the size of the hydrogel pad (50 mm^2^), CMC/PVA/CA_Ag030 and CMC/PVA/CA_Ag060 were non-hemolytic and slightly hemolytic, respectively. In this sense, as presented in Figure 10B, the HI was dependent on Ag content in the sample, as discussed for cytotoxicity in the previous Section 3.3.2, and for values of nanosilver below 9.5 μg, functionalized hydrogels are expected to be non-hemolytic.

## 4. Conclusions

In summary, this research presents the design, synthesis, and comprehensive characterization of innovative hybrid hydrogels made of carboxymethyl cellulose (CMC) and poly(vinyl alcohol) (PVA) chemically crosslinked by citric acid (CA) functionalized with silver nanoparticles (AgNPs) generated in situ through an environmental benign aqueous process. The results demonstrated that absorbent hybrid hydrogels were produced before and after incorporating AgNPs, with a typical swelling degree (SD) of 75–80% and a gel fraction (GF) higher than 98%. In addition, these polymeric hydrogel matrices presented hydrophilicity and permeability, compatible with wound dressing characteristics in keeping moisture balance and removing excess exudate from the diabetic wound site. The FTIR and XPS spectroscopic analyses associated with thermal profiles demonstrated that the chemical crosslinking mechanism of polymer chains was mainly related to the esterification reaction through carboxylic groups from CA with hydroxyl groups of CMC and PVA macromolecules forming covalent bonds. The esterification reaction produced a chemically crosslinked hybrid polymeric network, where no significant effect was detected upon functionalizing with AgNPs. The results assessed through in vivo assays demonstrated that different from hydrogels without nanosilver (i.e., 0% AgNPs), which showed high cytocompatibility and hemocompatibility, the hybrid polymer hydrogels functionalized with AgNPs displayed cytocompatibility and hemocompatibility dependent on the nanosilver concentration. In this view, based on the amalgamation of these biological responses (i.e., cytocompatibility and hemocompatibility) at the concentration limited to about 3.6 μg of AgNPs, the hybrid hydrogels would be suitable as wound dressings for assisting chronic diabetic wound healing. Moreover, as the pivotal property credited to the functionalization with AgNPs, the hybrids displayed antibacterial activity against four different reference strains, Gram-positive (*E. faecalis* and *S. aureus*) and Gram-negative (*E. coli* and *P. aeruginosa*), using the disk diffusion assay. This antibacterial behavior of the hybrids with embedded AgNPs was also confirmed using two representative strains of *E. coli* and *S. aureus* relying on bacteria growth inhibition assay. It should be highlighted that these are highly promising findings, considering that *S. aureus*, *E. coli*, and *P. aeruginosa* are among the most common microorganisms found in diabetic foot ulcers. Hence, relying on the results presented in this work and associated with future studies, it can be envisioned that these soft polymer-based hybrid platforms functionalized by silver nanoparticles may offer suitable tools for creating advanced dressings for potentially preventing and treating pathogenic bacterial infections of chronic diabetic wounds.

## Figures and Tables

**Table 1:** CMC/PVA/CA_AgNP nanocomposite hydrogel membranes.

Silver Nanocomposites Identification	CMC (m/v %)	PVA (m/v %)	[Ag]/ [CMC + PVA] m/m%	[CA]/ [CMC + PVA] m/m%	Mass of Ag/Nanocomposite Area (μg/cm^2^)
CMC/PVA/CA or CMC/PVA/CA_Ag0	80	20	0.00	25	0.0
CMC/PVA/CA_Ag015	80	20	0.15	25	7.5
CMC/PVA/CA_Ag030	80	20	0.30	25	15.0
CMC/PVA/CA_Ag060	80	20	0.60	25	30.0

## Data Availability

All relevant data are available in the manuscript or the supporting information.

## Supplementary Materials

The following supporting information can be downloaded at: https://www.mdpi.com/article/10.3390/polym15234542/s1, Table S1: Results of halo diameters (mm) of disk diffusion test performed according to Clinical and Laboratory Standards Institute using reference strains; Table S2: Quality control ranges for antimicrobials according to CLSI M100-28th ed.
