# Supplementary material for: Nanosilver-Functionalized Hybrid Hydrogels of Carboxymethyl Cellulose/Poly(Vinyl Alcohol) with Antibacterial Activity for Prevention and Therapy of Infections of Diabetic Chronic Wounds
Source: Polymers (Basel). 2023 Nov 27;15(23):4542. doi: 10.3390/polym15234542 (PMC10708083; doi:10.3390/polym15234542)
Supplement: Supplementary file 1 [file polymers-15-04542-s001.zip › polymers-2692828-supplementary.pdf]

polymers

MDPI

Supporting Information

Nanosilver Functionalized Hybrid Hydrogels of Carboxymethyl Cellulose/Poly(vinyl alcohol) with Antibacterial Activity for Prevention and Therapy of Infections of Diabetic Chronic Wounds

Nádia S. V. Capanema [1,#], Alexandra A. P. Mansur [1,#], Sandhra M. Carvalho [1], Talita Martins [1], Maysa S. Gonçalves [2], Rafaella S. Andrade [2], Elaine M. S. Dorneles [2], Letícia C. D. Lima [3], Érika L. F. C. de Alvarenga [4], Emanuel V. B. da Fonseca [4], Marcos Augusto de Sá [3], Andrey P. Lage [5], Zelia I. P. Lobato [5], Herman S. Mansur [1,*]

[1] Center of Nanoscience, Nanotechnology, and Innovation – CeNano²I, Department of Metallurgical and Materials Engineering, Federal University of Minas Gerais, UFMG, Belo Horizonte, Brazil
[2] Departamento de Medicina Veterinária, Universidade Federal de Lavras, UFLA, Lavras, Brazil
[3] Department of Morphology, Institute of Biological Sciences, Federal University of Minas Gerais, UFMG, Belo Horizonte, Brazil
[4] Department of Natural Sciences, Universidade Federal de São João Del-Rei, UFSJ, São João Del-Rei, Brazil
[5] Departamento de Medicina Veterinária Preventiva, Federal University of Minas Gerais, UFMG, Brazil
* Correspondence: hmansur@demet.ufmg.br
[#] These authors contributed equally to this work

Tables

Table S1. Results of halo diameters (mm) of disk diffusion test performed according to Clinical & Laboratory Standards Institute (CLSI) using reference strains ($\bar{x}$: Average; SD: standard deviation).

Antimicrobial	*E. faecalis* ATCC 29212 mm		*S. aureus* ATCC 29213 Mm		*E. coli* ATCC 25922 mm		*P. aeruginosa* ATCC 27853 mm	
	$\bar{x}$	SD	$\bar{x}$	SD	$\bar{x}$	SD	$\bar{x}$	SD
Cefotaxime (30 µg)	31.5	0.5	29.5	0.5	34.5	1.0	21.5	0.5
Norfloxacin (10 µg)	23.5	0.5	27.5	0.5	31.5	0.5	25.0	0.5

Table S2. Quality control ranges for antimicrobials according to CLSI M100-28th ed. [44].

Antimicrobial	*E. faecalis* ATCC 29212 mm	*S. aureus* ATCC 29213 mm	*E. coli* ATCC 25922 mm	*P. aeruginosa* ATCC 27853 mm
Cefotaxime (30 µg)	≥26	25-31	29-35	18-22
Norfloxacin (10 µg)	≥17	17-28	28-35	22-29
